# Performance of the Risk Scores for Predicting In-Hospital Mortality in Patients with Acute Coronary Syndrome in a Chinese Cohort

**DOI:** 10.31083/j.rcm2412356

**Published:** 2023-12-19

**Authors:** Lin Bai, Yi-Ming Li, Bo-Sen Yang, Yi-Heng Cheng, Yi-Ke Zhang, Guang-Zhi Liao, Yu-Yang Ye, Xue-Feng Chen, Hua Chai, Yong Peng

**Affiliations:** ^1^Department of Cardiology, West China Hospital, Sichuan University, 610041 Chengdu, Sichuan, China; ^2^Department of Academic Affairs, West China School of Medicine/West China Hospital, Sichuan University, 610041 Chengdu, Sichuan, China

**Keywords:** acute coronary syndrome, risk prediction, in-hospital mortality, GRACE, ACTION, CPACS

## Abstract

**Background:**

The prognosis of patients with acute coronary syndrome (ACS) varies greatly, and risk assessment models can help clinicians to identify and manage high-risk patients. While the Global Registry of Acute Coronary Events (GRACE) model is widely used, the clinical pathways for acute coronary syndromes (CPACS), which was constructed based on the Chinese population, and acute coronary treatment and intervention outcomes network (ACTION) have not yet been validated in the Chinese population.

**Methods:**

Patients with ACS who underwent coronary angiography or percutaneous coronary intervention from 2011 to 2020, were retrospectively recruited and the appropriate corresponding clinical indicators was obtained. The primary endpoint was in-hospital mortality. The performance of the GRACE, GRACE 2.0, ACTION, thrombolysis in myocardial infarction (TIMI) and CPACS risk models was evaluated and compared.

**Results:**

A total of 19,237 patients with ACS were included. Overall, in-hospital mortality was 2.2%. ACTION showed the highest accuracy in predicting discriminated risk (c-index 0.945, 95% confidence interval [CI] 0.922–0.955), but the calibration was not satisfactory. GRACE and GRACE 2.0 did not significantly differ in discrimination (*p* = 0.1480). GRACE showed the most accurate calibration in all patients and in the subgroup analysis of all models. CPACS (c-index 0.841, 95% CI 0.821–0.861) and TIMI (c-index 0.811, 95% CI 0.787–0.835) did not outperform (c-index 0.926, 95% CI 0.911–0.940).

**Conclusions:**

In contemporary Chinese ACS patients, the ACTION risk model’s calibration is not satisfactory, although outperformed the gold standard GRACE model in predicting hospital mortality. The CPACS model developed for Chinese patients did not show better predictive performance than the GRACE model.

## 1. Introduction

Acute coronary syndrome (ACS) is an unstable and progressive category within coronary heart disease (CHD), characterized by three serious and life-threatening clinical manifestations: ST-segment elevation myocardial infarction (STEMI), non-ST-segment elevation myocardial infarction (NSTEMI) and unstable angina (UA) [1, 2]. The clinical manifestations of ACS are broad, ranging from cardiac arrest and electrical or hemodynamic instability due to cardiogenic shock resulting from continuous ischemia or mechanical complications (e.g., severe mitral regurgitation) to patients without pain at the time of treatment [2]. Therefore, ACS management requires strict and scientific evaluation to identify high-risk patients [1, 2, 3].

The Global Registry of Acute Coronary Events (GRACE) is widely used as a risk assessment tool to predict predicting in-hospital mortality for patients with ACS, and has recently been updated to version 2.0 (GRACE 2.0) [1, 2, 4, 5]. However, it’s important to note that the GRACE risk scores were mainly developed in North America, South America, and Europe, with limited representation from Asian populations [4, 5]. Another notable ACS risk assessment tool is the thrombolysis in myocardial infarction (TIMI) risk score [6, 7]. This assessment model has been rigorously studied and independently shown to have a predictive effect on prognosis, as indicated by multivariate logistic regression analysis [6, 7]. The TIMI risk score is widely used in clinical practice due to its simplicity and ease of implementation. The acute coronary treatment and intervention outcomes network (ACTION) risk model has been recently developed and validated for ACS patient management [8, 9]. However, only a few studies have conducted external verification of this model [10]. Finally, a model from clinical pathways for acute coronary syndromes (CPACS) has been designed specifically for Chinese ACS patients [11]. Nonetheless, it’s worth noting that this scoring system lacks external validation, and its effectiveness beyond its original study remains unconfirmed.

The objective of this study was to assess the efficacy of five risk assessment models, GRACE, GRACE 2.0, ACTION, TIMI and CPACS, using data from a Chinese ACS cohort. Notably, this study represents the first external verification of the CPACS model since its creation and also the initial validation of ACTION for predicting in-hospital mortality in Chinese patients.

## 2. Methods

### 2.1 Study Population

For this study, we utilized the hospital information system of West China Hospital of Sichuan University to retrospectively enroll patients with acute coronary syndrome who underwent coronary angiography or percutaneous coronary intervention (PCI) in the Department of Cardiology from 2011 to 2020. All patients received treatment in accordance with the current American College of Cardiology/American Heart Association guidelines (**Supplementary Table 1**). We collected relevant clinical indicators are obtained from the patients’ medical history and laboratory examination during their hospital stay. The trial was approved by the Ethics Committee of West China Hospital of Sichuan University and registered at the Chinese Clinical Trial Registry, Chinese Clinical Trial Registry Number ChiCTR2100049313 (https://www.chictr.org.cn/indexEN.html).

### 2.2 Clinical End Points

The primary endpoint of interest was the risk sore performance evaluation/in-hospital mortality, which was defined as any postprocedural death within the same hospital admission.

### 2.3 Statistical Analysis

#### 2.3.1 Missing Data

To address missing data for clinical presentation and medical history variables, we imputed them as “no”. For the missing data related to the calculation of the risk model, we utilized the Missforest algorithm specific to the respective STEMI, NSTEMI or UA subpopulations to fill in the gaps.

#### 2.3.2 Data Analysis

Data analysis was performed using Excel 2019 (Microsoft, Redmond, WA, USA), SPSS 26 (IBM Corp., Armonk, NY, USA), STATA 16 (StataCorp LLC, College Station, TX, USA), MedCalc v19.6.1 (MedCalc Software, Ostend, Belgium) and GraphPad Prism 9.0 (GraphPad Software Inc., San Diego, CA, USA). Continuous data were expressed as mean ± standard deviation (SD), and ordinal or categorical variables were expressed as a percentage of counts and totals. We conducted a normal distribution test using the Kolmogorov‒Smirnov method on continuous data. Since all the test variables showed non-normal distribution, we used the Mann‒Whitney U test for comparing distributions between groups when dealing with two samples, and the Kruskal‒Wallis one-way ANOVA test for three samples. For comparing classification data, we utilized the Chi‒squared test, and in cases where the expected value was less than 5, Fisher’s exact test was employed.

#### 2.3.3 Risk Sore Model Calculation and Evaluation

This study utilized the latest versions of the GRACE, GRACE 2.0, ACTION, TIMI and CPACS risk scoring models to predict hospital mortality (Table 1) and conduct the performance calculation and evaluation [4, 5, 6, 7, 8, 11]. Individual patient scores were obtained by summarizing all relevant scoring variables weighted according to the model definition (**Supplementary Tables 2–7**). To analyze the discriminative performance of each risk model for in-hospital mortality, the receiver operating characteristic (ROC) curve with the area under the curve (AUC = c-index) was used as a cumulative measure. The c-index along with a 95% confidence interval (CI) was also reported. The DeLong method was used to compare the distinguishing performance between the models, with the assumption that results would be significantly different when the α probability was <0.05. By comparing the expected and observed events in the risk level (risk deciles of GRACE, GRACE 2.0, ACTION, TIMI and CPACS models), graphical analysis of the risk model calibration/goodness of fit was performed. Hosmer-Lemeshow goodness of fit test was also used to evaluate the calibration of the prediction model. Subgroup analyses were performed based on the ACS category (STEMI, NSTEMI or UA) and sex (male or female).

**Table 1.** **Characteristics of the risk models**.

Study Group	GRACE	GRACE 2.0	ACTION	TIMI		CPACS
Diagnostic Criteria for Entry	ACS	ACS	AMI	STEMI	NSTEMI/UA	ACS
Year of publication	2003; 2009	2014	2016	2000	2000	2017
Development cohort size	48,023; 62,935	32,037	243,440	141,114	7081	10,591
Mortality rate	4.6%		4.6%	6.7% (30 days)		3.2%
Discrimination performance (c-indices)	0.84		0.88	0.779	0.63	0.82 (male);
						0.78 (female)
Age	√	√	√	√	√	√
Sex						√
Weight				√		
Traditional cardiovascular factors				√	√	√
Angina				√	√	√
Pre-hospital medication history					√	√
Time to thrombolytic				√		
Cardiac arrest	√	√	√			
Cardiogenic shock			√			
Heart failure			√			
Heart rate	√	√	√	√		√
Killip	√	√		√		√
Systolic blood pressure	√	√	√	√		√
Diastolic blood pressure						√
ECG ST-segment changes	√	√	√	√	√	
Arrhythmia						√
Troponin levels	√	√	√		√	√
Kidney function	√	√	√			
Stent information					√	

√ denotes that the information was used for that scoring system. GRACE, Global Registry of Acute Coronary Events; CPACS, the clinical pathways for acute coronary syndromes; ACTION, acute coronary treatment and intervention outcomes network; TIMI, thrombolysis in myocardial infarction; ACS, acute coronary syndrome; AMI, acute myocardial infarction; (N)STEMI, (non) ST-segment elevation myocardial infarction; UA, unstable angina; ECG, electrocardiogram.

## 3. Results

### 3.1 Patient Characteristics

A total of 19,237 patients were diagnosed with ACS through coronary angiography were enrolled at West China Hospital of Sichuan University between January 1, 2011 and December 13, 2020. Among these patients, 7283 (37.9%) had STEMI, 4012 (20.9%) had NSTEMI and 7942 (41.3%) had UA.

The baseline characteristics of patients involved in risk score calculations are presented in Table 2 and **Supplementary Table 8**, categorized by ACS type and sex respectively.

**Table 2.** **Patient and procedural characteristics for all patients and subgroups based on acute coronary syndrome category**.

Characteristics			Total	STEMI	NSTEMI	UA	*p*
No. of patients			N = 19,237	N = 7283 (37.9)	N = 4012 (20.9)	N = 7942 (41.3)	
Age			64.49 ± 12.04	62.64 ± 13.14	66.03 ± 12.52	65.41 ± 10.58	<0.001
Male sex			14,635 (76.1)	5835 (80.1)	2986 (74.4)	5814 (73.2)	<0.001
Height			163.85 ± 7.41	164.44 ± 7.34	163.50 ± 8.08	163.49 ± 7.09	<0.001
Weight			65.35 ± 10.55	65.99 ± 10.51	64.91 ± 11.07	64.99 ± 10.29	<0.001
Medical history							
	Hypertension		10,680 (55.5)	3502 (48.1)	2332 (58.1)	4846 (61.0)	<0.001
	Diabetes mellitus		5264 (27.4)	1779 (24.4)	1275 (31.8)	2210 (27.8)	<0.001
	Hyperlipoproteinemia		1971 (10.2)	703 (9.7)	398 (9.9)	870 (11.0)	0.023
	Smoke		10,567 (54.9)	4442 (61.0)	2114 (52.7)	4011 (50.5)	<0.001
	Prior myocardial infarction		4257 (22.1)	1766 (24.2)	700 (17.4)	1791 (22.6)	<0.001
	Prior stroke or transient ischemic attacks		619 (3.2)	199 (2.7)	170 (4.2)	250 (3.1)	<0.001
	Family history of coronary heart disease		724 (3.8)	225 (3.1)	127 (3.2)	372 (4.7)	<0.001
	Previous antiplatelet agent use		9136 (47.5)	3760 (51.6)	1830 (45.6)	3546 (44.6)	<0.001
	Previous statin use		5386 (28.0)	1610 (22.1)	1129 (28.1)	2647 (33.3)	<0.001
	Symptoms of angina pectoris		5912 (30.7)	3406 (46.8)	1396 (34.8)	1110 (14.0)	<0.001
	Cardiac arrest		249 (1.3)	182 (2.5)	46 (1.1)	21 (0.3)	<0.001
	Shock		798 (4.1)	533 (7.3)	179 (4.5)	86 (1.1)	<0.001
At admission							
	Heart rate (beats/min)		77.30 ± 16.11	80.90 ± 18.53	78.50 ± 16.25	73.40 ± 12.35	<0.001
	Systolic blood pressure (mmHg)		128.31 ± 23.03	122.02 ± 24.32	129.60 ± 23.41	133.42 ± 20.05	<0.001
	Diastolic blood pressure (mmHg)		76.48 ± 14.30	75.60 ± 16.22	76.38 ± 14.32	77.33 ± 12.21	<0.001
	Killip class						<0.001
		I	16,968 (88.2)	5719 (78.5)	3379 (84.2)	7870 (99.1)	
		II	1224 (6.4)	833 (11.4)	361 (9.0)	30 (0.4)	
		III	331 (1.7)	213 (2.9)	109 (2.7)	9 (0.1)	
		IV	714 (3.7)	518 (7.1)	163 (4.1)	33 (0.4)	
	ST elevation or depression		7028 (36.5)	5072 (69.6)	1059 (26.4)	897 (11.3)	<0.001
	Arrhythmia		7514 (39.1)	5148 (70.7)	1186 (29.6)	1180 (14.9)	<0.001
	TPN-T		1386.63 ± 2595.71	2926.91 ± 3373.06	1171.35 ± 1899.75	82.91 ± 407.13	<0.001
	CERA		1.07 ± 0.84	1.07 ± 0.78	1.21 ± 1.16	1.01 ± 0.68	<0.001
	Coronary artery blockage ≥50%		14,248 (74.1)	6103 (83.8)	3002 (74.8)	5143 (64.8)	<0.001
In-hospital mortality			414 (2.2)	288 (4.0)	103 (2.6)	23 (0.3)	<0.001

(N)STEMI, (non) ST-segment elevation myocardial infarction; CERA, creatinine; TPN-T, cardiac troponin-T; UA, unstable angina.

### 3.2 Clinical Outcomes

In this cohort, there were a total of 414 patients who experienced endpoint events, resulting in an in-hospital mortality rate of 2.2%. Upon conducting subgroup analysis based on ACS category, we observed varying incidence rates among different groups: STEMI patients had the highest incidence at 4.0%, followed by NSTEMI patients at 2.6%, and UA patients had the lowest incidence at 0.3% (*p* < 0.0001). Additionally, when considering sex as a factor, we found that female patients (2.7%) had a slightly higher incidence of events compared to male patients (2.0%) (*p* = 0.002).

### 3.3 Risk Model Performance Evaluation

Characteristics of the GRACE, GRACE 2.0, ACTION, TIMI and CPACS risk scoring models for the prediction of in-hospital mortality are reported in Table 2.

#### 3.3.1 Missing Data

Missing data for clinical presentation and medical history variables were imputed as “no”. These variables included smoking history (n = 212) and heart failure performance (n = 575). For the missing variables used in the five scoring systems, such as weight (n = 2925), systolic blood pressure (n = 23), diastolic blood pressure (n = 27), heart rate (n = 6), creatinine (n = 61) and troponin (n = 135), we applied the Missforest algorithm for imputation.

#### 3.3.2 Risk Model Discrimination

**Fig. 1.**
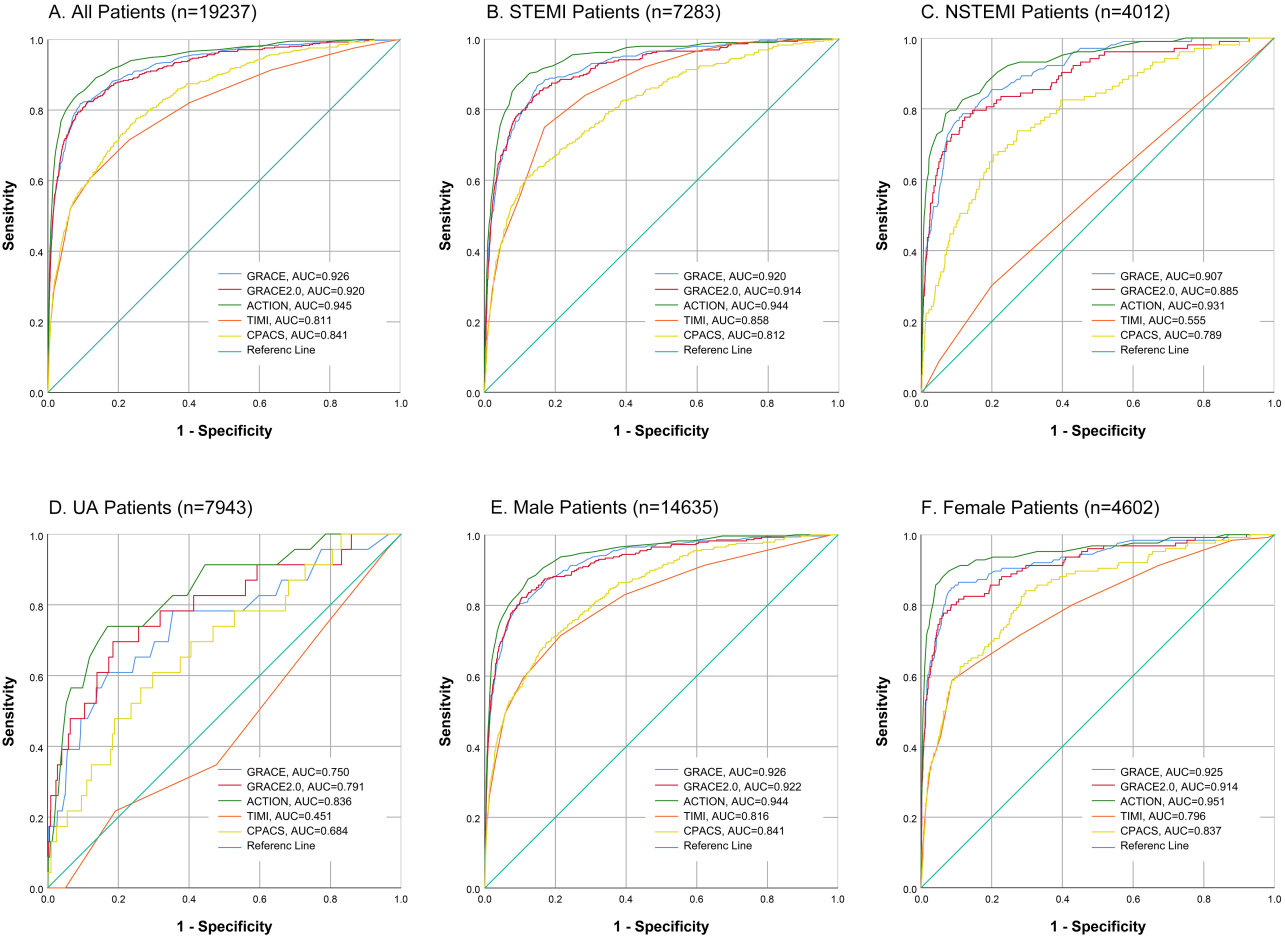
**Discrimination for the five risk models**. Analysis of the comparative risk model discrimination performance for in-hospital mortality in patients with ACS was conducted using ROC curves of five risk models: GRACE, GRACE 2.0, ACTION, TIMI and CPACS. The evaluation included all patients with ACS (A), as well as specific subgroups including those with STEMI (B), NSTEMI (C), UA (D), male (E) and female (F). (N)STEMI, (non) ST-segment elevation myocardial infarction; UA, unstable angina; GRACE, Global Registry of Acute Coronary Events; CPACS, the clinical pathways for acute coronary syndromes; ACS, acute coronary syndromes; ACTION, acute coronary treatment and intervention outcomes network; TIMI, thrombolysis in myocardial infarction; AUC, the area under the curve.

For the ACS population, all five risk score models exhibited good discrimination, with c-index values ranging from 0.811 (TIMI) to 0.945 (ACTION). Among all five models, ACTION performed most accurately with a c-index of 0.945 (95% CI 0.922–0.955) (Fig. 1A). There were no significant differences between GRACE and GRACE 2.0 (*$p_{\text{GRACE }\text{ 𝑣𝑠}\text{. GRACE 2.0}}$* = 0.1480). However, the pairwise comparisons of GRACE or GRACE 2.0 and the other three risk models showed significant differences (Table 3).

**Table 3.** **Risk model discrimination and calibration performance for all patients and subgroups based on acute coronary syndrome category**.

Characteristics		Total	STEMI	NSTEMI	UA
No. of patients		N = 19,237	N = 7283 (37.9)	N = 4012 (20.9)	N = 7942 (41.3)
In-hospital mortality		414 (2.2)	288 (4.0)	103 (2.6)	23 (0.3)
Risk model discrimination					
	GRACE	0.926 (0.911–0.940)	0.920 (0.903–0.937)	0.907 (0.879–0.935)	0.750 (0.635–0.865)
	GRACE 2.0	0.920 (0.905–0.935)	0.914 (0.896–0.933)	0.885 (0.848–0.923)	0.791 (0.685–0.897)
	ACTION	0.945 (0.933–0.957)	0.944 (0.930–0.959)	0.931 (0.904–0.957)	0.836 (0.749–0.923)
	TIMI	0.811 (0.787–0.835)	0.858 (0.837–0.879)	0.555 (0.496–0.613)	0.451 (0.333–0.570)
	CPACS	0.841 (0.821–0.861)	0.812 (0.785–0.840)	0.789 (0.743–0.835)	0.684 (0.574–0.783)
Statistical comparison					
	GRACE *vs*. GRACE 2.0	*p* = 0.1480	*p* = 0.1745	*p* = 0.0478	*p* = 0.0993
	GRACE *vs*. ACTION	*p* = 0.0004	*p* = 0.0002	*p* = 0.0786	*p* = 0.1064
	GRACE *vs*. TIMI	*p * < 0.0001	*p * < 0.0001	*p * < 0.0001	*p* = 0.0178
	GRACE *vs*. CPACS	*p * < 0.0001	*p * < 0.0001	*p * < 0.0001	*p* = 0.1010
	GRACE 2.0 *vs*. ACTION	*p * < 0.0001	*p* = 0.0001	*p* = 0.0031	*p* = 0.3165
	GRACE 2.0 *vs*. TIMI	*p * < 0.0001	*p * < 0.0001	*p * < 0.0001	*p* = 0.0042
	GRACE 2.0 *vs*. CPACS	*p * < 0.0001	*p * < 0.0001	*p * < 0.0001	*p* = 0.0006
	ACTION *vs*. TIMI	*p * < 0.0001	*p * < 0.0001	*p * < 0.0001	*p* = 0.0007
	ACTION *vs*. CPACS	*p * < 0.0001	*p * < 0.0001	*p * < 0.0001	*p* = 0.0030
	TIMI *vs*. CPACS	*p* = 0.0077	*p* = 0.0001	*p * < 0.0001	*p* = 0.1589
Risk model calibration – Mean risk prediction					
	GRACE	2.15 ± 7.55	3.95 ± 10.66	2.57 ± 7.05	0.29 ± 0.50
	GRACE 2.0	2.15 ± 7.99	3.95 ± 11.03	2.57 ± 7.68	0.29 ± 1.11
	ACTION	2.15 ± 8.52	3.95 ± 11.98	2.57 ± 8.91	0.29 ± 0.75
	TIMI	2.15 ± 4.51	3.95 ± 7.28	2.57 ± 0.51	0.29 ± 0.05
	CPACS	2.15 ± 4.22	3.95 ± 6.17	2.57 ± 3.72	0.29 ± 0.26
Risk model calibration – Hosmer-Lemeshow					
	GRACE	*p* = 0.359	*p* = 0.292	*p* = 0.483	*p* = 0.316
	GRACE 2.0	*p * < 0.001	*p * < 0.001	*p* = 0.007	*p* = 0.057
	ACTION	*p* = 0.013	*p* = 0.009	*p* = 0.914	*p* = 0.856
	TIMI	*p* = 0.508	*p* = 0.006	*p* = 0.765	*p* = 0.178
	CPACS	*p* = 0.148	*p* = 0.034	*p* = 0.863	*p* = 0.857

(N)STEMI, (non) ST-segment elevation myocardial infarction; UA, unstable angina; GRACE, Global Registry of Acute Coronary Events; CPACS, the clinical pathways for acute coronary syndromes; ACTION, acute coronary treatment and intervention outcomes network; TIMI, thrombolysis in myocardial infarction.

The subgroup analysis based on ACS category is shown in Fig. 1B–D and Table 3. All five models show good model discrimination in STEMI patients, with the c-index ranging from 0.812 (CPACS) to 0.944 (ACTION). The discrimination performance of GRACE (c-index 0.920, 95% CI 0.903–0.937) and GRACE 2.0 (c-index 0.914, 95% CI 0.896–0.933) models did not show a significant difference (*$p_{\text{GRACE }\text{𝑣𝑠}\text{. GRACE 2.0}}$* = 0.1745). In NSTEMI patients, the ACTION model (c-index 0.931, 95% CI 0.904–0.957) demonstrated the best performance, while the TIMI model (c-index 0.555, 95% CI 0.496–0.613) performed poorly. For UA patients, the performance of all five prediction models was unsatisfactory. The ACTION model (c-index 0.836, 95% CI 0.749–0.923) showed the best discrimination, while the c-index of TIMI (c-index 0.451, 95% CI 0.333–0.570) was even lower than 0.5. No significant difference was observed between the GRACE, GRACE 2.0 and ACTION models.

Since the CPACS model conducts analysis based on sex, we also verified that aspect of the prognostic ability. The subgroup analysis according to sex is shown in Fig. 1E,F and **Supplementary Table 9**. No significant difference was observed between GRACE and GRACE 2.0 in either the male or female subgroups. In male patients, all five models demonstrated good discrimination, with c-index values ranging from 0.816 (TIMI) to 0.944 (ACTION). For female patients, the discrimination performance of the TIMI (c-index 0.796, 95% CI 0.750–0.841) and CPACS (c-index 0.837, 95% CI 0.799–0.874) models did not show a significant difference (*$p_{\text{TIMI }\text{𝑣𝑠}\text{. CPACS}}$* = 0.0932). Among all five models, ACTION performed well, with a c-index of 0.951 (95% CI 0.926–0.975).

#### 3.3.3 Risk Model Calibration

**Fig. 2.**
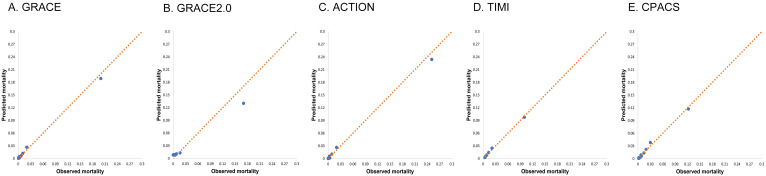
**Calibration for the five risk models**. Risk model calibration for GRACE (A), GRACE 2.0 (B), ACTION (C), TIMI (D) and CPACS (E) risk models, comparing observed and predicted mortality in risk quintiles of all patients with acute coronary syndrome. GRACE, Global Registry of Acute Coronary Events; CPACS, the clinical pathways for acute coronary syndromes; ACTION, acute coronary treatment and intervention outcomes network; TIMI, thrombolysis in myocardial infarction.

The calibration/goodness of fit of the risk models was evaluated using graphical analysis (Fig. 2 and **Supplementary Figs. 1,2**) and Hosmer-Lemeshow goodness of fit test (Table 3 and **Supplementary Table 9**) for all ACS patients and subgroups. For all ACS patients, the Hosmer-Lemeshow goodness of fit test indicates that GRACE (*p* = 0.359), TIMI (*p* = 0.508) and CPACS (*p* = 0.148) fit the data well, with no significant differences between the observational data and forecast data. However the performance of GRACE 2.0 (*p* < 0.001) and ACTION (*p* = 0.013) were unsatisfactory.

The graphical analysis of the risk model calibration/goodness of fit for all ACS patients also indicated that GRACR, TIMI, and CPACS exhibited more accurate calibration. In contrast, GRACE 2.0 and ACTION underestimated the mortality risk of high-risk patients in our cohort, while GRACE 2.0 overestimated the mortality risk of low-risk patients (Fig. 2).

The subgroup analysis based on ACS category is presented in Table 3 and **Supplementary Fig. 1**. Among STEMI patients, only GRACE (*p* = 0.292) showed accurate calibration, while GRACE 2.0, ACTION and CPACS all underestimated the probability of death in high-risk patients, and TIMI overestimated the probability of death (**Supplementary Fig. 1A**). For NSTEMI patients, GRACE 2.0 significantly underestimated the mortality risk of high-risk patients and overestimated the mortality risk of low-risk patients. Although the Hosmer-Lemeshow goodness of fit test showed that all five models fit the data of UA patients well, the graphical analysis of the risk model calibration/goodness of fit appeared slightly scattered.

Regarding the subgroup analysis based on sex, the results of the Hosmer-Lemeshow goodness of fit test and graphical analysis of the risk model calibration/goodness of fit are presented in **Supplementary Table 9** and **Supplementary Fig. 2**. In male patients, all four models, except GRACE 2.0 fit the data well, while GRACE (*p* = 0.005), GRACE 2.0 (*p* = 0.008) and ACTION (*p* = 0.016) did not show a good fit between the model and the data in female patients.

## 4. Discussion

Here, we presented a comparative performance evaluation between the GRACE, GRACE 2.0, ACTION, TIMI and CPACS risk models for predicting in-hospital mortality in a Chinese cohort with ACS. This is also the first external verification of the CPACS model since its establishment and the first verification of ACTION to predict in-hospital mortality in Chinese patients. Our study found that the ACTION model demonstrated the best discrimination in all five risk scores among ACS patients and in the ACS subgroup analysis of gender. However, the calibration was not satisfactory. GRACE demonstrated proper discrimination and calibration across all classifications. While GRACE and GRACE 2.0 did not show significant differences in discrimination (*$p_{\text{GRACE }\text{𝑣𝑠}\text{. GRACE 2.0}}$* = 0.1480), the calibration of GRACE 2.0 was unsatisfactory. Neither CPACS (c-index 0.841, 95% CI 0.821–0.861) and TIMI (c-index 0.811, 95% CI 0.787–0.835) exhibited better performance compared to GRACE. However, GRACE displayed the most accurate calibration for all patients and in the subgroup analysis of all models. Finally, CPACS performed well except in STMEI patients.

The GRACE study is currently the world’s first prospective prediction study of all types of unscreened ACS patients conducted in multiple countries [4, 12]. Extensive external verifications have confirmed the value of GRACE in assessing early and delayed invasive management strategies in ACS, and it has been recommended by the ESC and AHA guidelines [1, 2, 10, 13, 14, 15, 16, 17]. The updated version of the GRACE 2.0 model, released in 2016, introduced nonlinear association and improved bedside ease of use through mobile phone software [5]. In our study, both GRACE and GRACE 2.0 performed excellently in model discrimination. They effectively distinguished the related risks of in-hospital death of ACS patients, and no significant difference was found between them. However, the calibration of GRACE 2.0 was poor, and it significantly underestimates the risk of death for high-risk patients. Among the five risk assessment models included in this study, only the GRACE score showed a good degree of calibration in the STEMI subgroup analysis.

The TIMI risk score is a clinical risk score for the prognosis of patients with ACS. The variables in this score are derived from an independent predictive effect on the prognosis, identified through multivariate logistic regression analysis in the TIMI trial population [6, 7]. Different scoring systems are available based on the ACS disease spectrum, including separate scores for STMEI and UA/NSTEMI patients. These scores primarily rely on data from electrocardiograms and clinical characteristics, making them simple and easily obtainable, making them suitable for use in emergency departments. The TIMI score has been validated to effectively stratify high-risk patients with chest pain and predict the incidence of short-term and long-term adverse cardiovascular events [18, 19]. However, the performance of the TIMI score was not satisfactory in our data. Despite proper calibration, the discrimination of the model is inferior to other models. This discrepancy may arise because the scoring system does not include certain factors that are relevant to poor prognosis in myocardial injury. The accuracy of the model is reduced by not considering cardiac biomarkers and ST-segment resolution for STEMI patients, and blood pressure and heart rate for UA/NSTEMI patients.

ACTION Registry- Get With The Guidelines is a voluntary, hospital-based registry system that receives data from consecutive acute myocardial infarction (AMI) patients from participating hospitals across the United States, including STEMI or NSTEMI. The ACTION risk model for in-hospital mortality, based on the registry, was developed from 65,668 patients with AMI and was updated in 2016 to include patients from 2012 to 2013 [8, 9]. The model has been externally validated in a Spanish and a German cohort [10, 20]. In the Spanish cohort, both ACTION and GRACE showed proper distinctions between in-hospital deaths (C statistics were 0.90 and 0.90, respectively) and had good calibration (Hosmer-Lemeshow goodness of fit test values 0.50 and 0.47, respectively) [20]. In the German cohort, Parco *et al*. [10] evaluated the predictive efficacy of the ACTION model, including 1567 (non) ST-segment elevation myocardial infarction patients who received invasive treatment at Düsseldorf University Hospital in Germany from 2014 to 2018. The results showed that the performance of the ACTION and GRACE risk models are comparable (c-index 0.84, *$p_{\text{GRACE }\text{𝑣𝑠}\text{. ACTION}}$* = 0.68), with an advantage in for the ACTION model in NSTEMI patients (c-index 0.87 [ACTION] *vs*. 0.84 [GRACE]; *$p_{\text{GRACE }\text{𝑣𝑠}\text{. ACTION}}$* = 0.02) [10]. A key distinguishing feature of ACTION from TIMI and GRACE scores is its ability to better differentiate high-risk patients when included after cardiac arrest, in cardiogenic shock, and in HF. In this Chinese cohort, the ACTION model not only showed the best discrimination among ACS patients with a c-index of 0.945, but also performed best in the subgroup analysis of ACS category or gender. However, the ACTION model is not properly calibrated for all ACS patients (*p* = 0.013), STEMI patients (*p* = 0.009), and female patients (*p* = 0.016), this is mainly due to underestimating the risk of death in high-risk groups. Another noteworthy point is that although the target population of the ACTION score is AMI patients, in the subgroup analysis of UA patients, the action score still showed good differentiation and calibration, which may be caused by the lower mortality of the UA subgroup.

The CPACS program is a quality improvement program conducted by the Chinese Heart Association, focusing on the management of inpatients suspected of ACS patients [21]. The subsequent Acute Coronary Syndrome Clinical Pathway-Phase 2 (CPACS-2) evaluated the effectiveness of interventions based on the clinical path for managing ACS patients in 75 hospitals in China [22]. The CPACS risk scoring model, designed to assess in-hospital mortality risk, was initially developed for different sexes among 6790 patients who were hospitalized for suspected acute coronary syndrome [11]. It was later compared to the GRACE risk score in predicting in-hospital mortality risk among 3801 patients [11]. Prior to this study, there was no external verification of the CPACS model.

Although CPACS is an in-hospital mortality risk assessment model for ACS patients established using a Chinese population, it did not perform better than the GRACE risk score in our cohort, either for all ACS patients (c-index 0.841 *vs*. 0.926; *$p_{\text{GRACE }\text{𝑣𝑠}\text{. CPACS}}$* < 0.001) or for sex subgroups (c-index for male 0.841 *vs*. 0.926; *$p_{\text{GRACE }\text{𝑣𝑠}\text{. CPACS}}$* < 0.001; c-index for female 0.837 *vs*. 0.925; *$p_{\text{GRACE }\text{𝑣𝑠}\text{. CPACS}}$* < 0.0001). This may be due to the relatively small number of patients included in the construction of the CPACS model. Additionally, some medical history information, such as diabetes and prehospital medication history, may not have been obtained in time in critically ill patients. However, CPACS (*p* = 0.113) had better calibration in female patients than GRACE (*p* = 0.005).

In recent years, an increasing number of risk scores have been used to predict the in-hospital and long-term risk of ACS patients. Among these factors, The History, Electrocardiogram, Age, Risk factor, and Troponin (HEART) Score is frequently utilized in emergency departments or chest pain centers, where clinical data are limited, and urgent diagnosis and further management are needed [23]. Other scores, such as the Vancouver Chest Pain Rules (VCPR), the North American Chest Pain Rule (NACPR), the Emergency Department Assessment of Chest Pain Score (EDACS) tools, the Manchester Acute Coronary Syndromes (MACS), and the Troponin-only Manchester Acute Coronary Syndromes (T-MACS) decision aids, can be chosen based on local standards of care and provider risk tolerance [24]. Notably, in patients with AMI, the Killip classification performed at admission is a simple and useful clinical marker of high risk for early and late adverse cardiovascular events [25]. TIMI risk scores also tend to facilitate quick early ratings. As a widely used ACS risk assessment model, the GRACE score has demonstrated good predictive performance in hospital events and 6-month follow-up. However, with advancements in test items and treatment methods, further investigation is needed to assess predictive sensitivity and accuracy. Given the changes in interventions and decision points in recent years, evaluating modern data would be particularly beneficial. The ACTION score may be most useful for severely ill patients and provide guidance for new interventions [26]. Considering that female patients often have more cardiac risk factors than male patients, even though they are low-risk populations in most cases [27], attention should be given to risk models that consider gender divisions, such as CPACS. In summary, at present many risk models have applicable scenarios and limitations, but the continuity among different risk models is not strong. Therefore, a dynamic risk assessment based on time and gender seems to be more suitable for patients during treatment, and this may be a direction of the development for future risk assessment models.

Some factors have not yet been added to current ACS risk assessment models, but significantly impact patient prognosis. Chronic kidney diseases patients have a higher risk of ischemia and AMI and a worse prognosis, especially in dialysis patients. Both cardiac and renal insufficiency can aggravate the prognosis and lead to disease progression [28]. Type 2 diabetes is another critical factor, increasing the risk of major adverse cardiovascular events. Notably, the bleeding tendency associated with diabetes appears to be limited to patients receiving insulin therapy [29]. A new algorithm called SCORE2-Diabetes has been developed to predict 10-year cardiovascular disease (CVD) risk in patients with type 2 diabetes, helping to identify individuals at high CVD risk. The algorithm utilizes sex-specific competing risk-adjusted models, incorporating traditional risk factors (e.g., age, smoking, systolic blood pressure, total cholesterol, and high-density lipoprotein cholesterol) and diabetes-related variables (e.g., age at diabetes diagnosis, glycated hemoglobin, and creatinine-based estimated glomerular filtration rate) [30]. Patients with both standard modifiable cardiovascular risk factors and prior CVD have a higher mortality regardless of their sex [31]. Therefore, including coexisting conditions in long-term risk assessment models may be important. Additionally, myocardial infarction without obstructive coronary artery disease is a significant consideration, as it accounts for a considerable proportion of AMI cases and has received extensive attention in recent years [32]. However, the current scoring system lacks a corresponding assessment for this condition.

The development of cardiac imaging has benefited from the advances in Computed Tomography technology. The emergence of coronary computed tomography angiography (CCTA) has revolutionized the non-invasive evaluation and rapid risk stratification of coronary heart disease. Intact fibrous cap ACS patients exhibit a unique inflammatory response and a lower MACE risk compared to rupture of the fibrous cap ACS patients [33]. By conducting a comprehensive evaluation based on CCTA, considering multiple aspects such as stenosis degree, plaque characteristics, and functional reserve, clinicians can obtain vital information for accurate prognosis stratification of patients [34]. Leveraging radiomics and machine learning techniques, CCTA images can be objectively and mathematically evaluated, providing enhanced precision analysis. In the era of big data analysis and artificial intelligence, CCTA is poised to perform multi-dimensional risk stratification of patients with coronary heart disease [34].

This research does has some limitations, including the following: (1) There are still missing data points, such as 2925 missing values for weight. Although the random forest algorithm was used to supplement the missing values, the potential impact on the results is still unknown. (2) The c-index of the risk models in this study is high, with the highest being 0.945. This may have been influenced by the lower incidence of death events in the cohort itself and the large number of low-risk individuals. Higher negative sample populations can improve the model’s performance by affecting the negative predictive value. (3) The lower mortality observed in the ACS patients included in this study may attributed to the higher diagnostic rate of UA in China. The overestimated proportion of UA in China may be due to physicians’ tendency to make a diagnosis based on the clinical manifestations during the first visit without considering dynamic changes in high-sensitivity troponin and ECG. This proportion is similar to that found in a previously published Chinese multicenter ACS cohort study (CPACS registry, UA >40%) [11, 21].

## 5. Conclusions

In contemporary Chinese ACS patients with, the ACTION risk model outperforms the gold standard GRACE model in predicting hospital mortality, despite its unsatisfactory calibration. The CPACS model developed for Chinese patients did not exhibit better predictive performance than the GRACE model. Nevertheless, the GRACE model continues to demonstrate a strong performance across all aspects and remains a reliable tool for ACS risk prediction for the foreseeable future.

## Data Availability

The datasets used and analyzed during the current study are available from the corresponding author on reasonable request.

## Abbreviations

ACS, acute coronary syndrome; ACTION, acute coronary treatment and intervention outcomes network; AUC, Area under the curve; CHD, coronary heart disease; CI, confidence interval; CPACS, clinical pathways for acute coronary syndromes; GRACE, Global Registry of Acute Coronary Events; NSTMEI, non-ST-segment elevation myocardial infarction; PCI, percutaneous coronary intervention; ROC, receiver operating characteristic; STEMI, ST-segment elevation myocardial infarction; TIMI, thrombolysis in myocardial infarction; UA, unstable angina.

## Supplementary Material

Supplementary material associated with this article can be found, in the online version, at https://doi.org/10.31083/j.rcm2412356.
