# Supplementary material for: Performance of the Risk Scores for Predicting In-Hospital Mortality in Patients with Acute Coronary Syndrome in a Chinese Cohort
Source: Rev Cardiovasc Med. 2023 Dec 19;24(12):356. doi: 10.31083/j.rcm2412356 (PMC11272866; doi:10.31083/j.rcm2412356)
Supplement: Supplementary file 1 [file 2153-8174-24-12-356-s1.zip › 2153-8174-24-12-356-s1.pdf]

SUPPLEMENTAL MATERIAL

This appendix has been provided by the authors to give readers additional about their work.

Table of contents

Appendix I. Supplementary Tables

Page 2. Supplementary Table S1. In-hospital management strategies for patients at different time periods.

Page 3. Supplementary Table S2. GRACE in-hospital mortality risk model parameters and point assignment.

Page 4. Supplementary Table S3. GRACE2.0 in-hospital mortality risk model parameters and point assignment.

Page 5. Supplementary Table S4. TIMI in-hospital mortality risk model parameters and point assignment for STEMI patients.

Page 6. Supplementary Table S5. TIMI in-hospital mortality risk model parameters and point assignment for NSTEMI/UA patients.

Page 7. Supplementary Table S6. ACTION in-hospital mortality risk model parameters and point assignment.

Page 8. Supplementary Table S7. CPACS in-hospital mortality risk model parameters and point assignment.

Page 9. Supplementary Table S8. Patient and procedural characteristics for all patients and subgroups based on gender.

Page 10. Supplementary Table S9. Risk model discrimination and calibration performance for all patients and subgroups based on gender.

Appendix II. Supplementary Figures

Page 10. Supplementary Figure S1. Discrimination for the five risk models in subgroup analysis based on acute coronary syndrome category.

Page 11. Supplementary Figure S2. Discrimination for the five risk models in subgroup analysis based on gender.

Supplementary Table S1. In-hospital management strategies for patients at different time periods.

	STEMI			NSTEMI/UA		
	2011-2013	2013-2015	2015-2020	2011-2012	2012-2014	2014-2020
Reperfusion therapy	1. All patients with history of chest pain/discomfort of <12h and with persistent ST-segment elevation or (presumed) new left bundle-branch block.<br>2. There is clinical and/or ECG evidence of ongoing ischemia even if, according to patient, symptoms started >12 h before.<br>3. In stable patients presenting >12 to 24 h after symptom onset.	1. Ischemic symptoms < 12h<br>2. Ischemic symptoms <12h and contraindications to fibrinolytic therapy irrespective of time delay from FMC.<br>3. Cardiogenic shock or acute severe HF irrespective of time delay from MI onset<br>4. Evidence of ongoing ischemia 12 to 24 h after symptom onset.<br>5. PCI should not be performed in a noninfarct artery at the time of primary PCI in patients with STEMI who are hemodynamically stable.	PCI of a noninfarct artery may be considered in selected patients with STEMI and multivessel disease who are hemodynamically stable, either at the time of primary PCI or as a planned staged procedure.	1. An early invasive strategy is indicated in UA/NSTEMI patients who have refractory angina or hemodynamic or electrical instability.<br>2. An early invasive strategy is indicated in initially stabilized UA/NSTEMI patients who have an elevated risk for clinical events.	1. An early invasive strategy is indicated in UA/NSTEMI patients who have refractory angina or hemodynamic or electrical instability.<br>2. An early invasive strategy is indicated in initially stabilized UA/NSTEMI patients who have an elevated risk for clinical events.<br>3. It is reasonable to choose an early invasive strategy over a delayed invasive strategy for initially stabilized high-risk patients with UA/NSTEMI. For patients not at high risk, a delayed invasive approach is also reasonable.	1. An urgent/immediate invasive strategy is indicated in patients with NSTE-ACS who have refractory angina or hemodynamic or electrical instability.<br>2. An early invasive strategy is indicated in initially stabilized patients with NSTE-ACS who have an elevated risk for clinical events.<br>3. It is reasonable to choose an early invasive strategy over a delayed invasive strategy for initially stabilized high-risk patients with NSTE-ACS. For those not at high/intermediate risk, a delayed invasive approach is reasonable.
Stent	It is reasonable to use a DES as an alternative to a BMS for primary PCI	Placement of a stent (BMS or DES) is useful in primary PCI	DES	BMS/DES	BMS/DES	DES
Antiplatelet therapy	Aspirin + Clopidogrel	Aspirin + Clopidogrel	Aspirin + Clopidogrel/ Aspirin + Ticagrelor	Aspirin + Clopidogrel	Aspirin + Clopidogrel	Aspirin + Clopidogrel/ Aspirin + Ticagrelor
Estimation of Level of Risk	Risk factors	TIMI<br>GRACE	TIMI<br>GRACE	Risk factors	TIMI<br>GRACE	TIMI<br>GRACE

Supplementary Table S2. GRACE in-hospital mortality risk model parameters and point assignment.

Killip Class	Point	Systolic BP	Point	Heart Rate	Point	Age	Point	Creatinine	Point	Baseline risk factors	Point
I	0	≤70	66	≤70	10	≤30	0	0-0.39	3	Cardiac Arrest at Admission	38
II	17	70-89	53	70-89	15	30-49	10	0.4-0.9	9	ST-Segment Deviation	18
III	34	90-109	40	90-109	26	50-69	29	1.0-1.9	32	Positive cardiac markers	14
IV	51	110-129	27	110-129	32	70-79	56	≥2	51	STEMI	14
		≥130	19	130-149	24	80-89	73				
				150-169	16	≥90	91				
				170-199	8						
				≥200	0						

Risk model parameters and point assignment of the GRACE risk model for prediction of in-hospital mortality(1). BP = Blood pressure; STEMI = ST-segment elevation myocardial infarction.

Supplementary Table S3. GRACE2.0 in-hospital mortality risk model parameters and point assignment.

Intercept	-7.7035
AGE (per 1 yr)	0.0531
PULSE (per 1 BPM)	0.0087
SYSTOLIC BLOOD PRESSURE (per 1 mmHG)	-0.0168
INITIAL SERUM CREATININE, mg,dl	0.1823
KILLIP CLASS (1,2,3, or 4)	0.6931
CARDIAC ARREST at presentation*	1.4586
INITIAL CARDIAC ENZYME Positive*	0.4700
ST SEGMENT DEVIATION*	0.8755

* Enter a value of 1 if factor is present, 0 otherwise.

To obtain estimated risk of death from above estimates

Compute XB, where X=individual patient's value for each factor (eg, age=57, pulse=70…), and B=estimates above, including the intercept.

XB is then the summed product of the patient characteristics times the estimates, with the intercept added for every patient.

The probability of in-hospital death is then

P = (Exp**XB)/(1+exp**(XB)) (= .21693),

where exp is 2.71828…, and ** means raised to that power (XB power).

Supplementary Table S4. TIMI in-hospital mortality risk model parameters and point assignment for STEMI patients.

**Historical**	
Age 65-74	**2**
≥ 75	**1**
DM/HTN or angina	**1**
**Exam**	
SBP < 100	**3**
HR > 100	**2**
Killip II- IV	**2**
Weight < 67 kg	**1**
**Presentation**	
Anterior STE or LBBB	**1**
Time to rx > 4 hrs	**1**
**Riske sore = Total**	**(0-14)**

TIMI Risk Score for STEMI summarized for printing on laminated card for clinical use. DM indicates diabetes mellitus; STEMI, ST elevation myocardial infarction; SBP, systolic blood pressure; HR, heart rate; and rx, treatment.

Supplementary Table S5. TIMI in-hospital mortality risk model parameters and point assignment for NSTEMI/UA patients.

Characteristics	Sores
Age, ≥65y	1
At least 3 risk factors for CAD*	1
Significant coronary stenosis (eg, prior coronary stenosis ≥50%)	1
ST deviation	1
Severe anginal symptoms (eg, ≥2 anginal events in last 24h)	1
Use of aspirin in last 7 days	1
Elevated serum cardiac markers	1

CAD indicates coronary artery disease; UA, unstable angina; NSTEMI, non-ST elevation myocardial infarction.

* Risk factors included family history of CAD, hypertension, hypercholesterolemia, diabetes, or being a current smoker.

Supplementary Table S6. ACTION in-hospital mortality risk model parameters and point assignment.

Age	Pts	SBP	Pts	CrCl	Pts	Cardiac Arrest	Pts	Shock	Pts	Heart Rate	Pts	Heart Failure	Pts	STEMI	Pts	Troponin	Pts
<40	0	>200	0	≥90	0	No	0	No	0	≤40	0	No	0	No	0	<10	0
40-49	3	181-200	3	60-<90	4	Yes	14	Yes	13	41-60	1	Yes	5	Yes	5	10-<20	1
50-59	7	170-180	5	45-<60	8					61-70	2					20-<30	2
60-69	10	161-170	7	30-<45	11					71-80	3					≥30	3
70-79	13	151-160	9	<30 or Dialysis	15					81-100	4						
80-89	17	131-150	11							101-110	5						
>90	20	121-130	13							111-130	7						
		111-120	15							131-150	8						
		91-110	16							>150	9						
		≤90	19														

Supplementary Table S7. CPACS in-hospital mortality risk model parameters and point assignment.

Effect factors*	Beta-coefficients	
	Backwards selection—men	Backwards selection—women
Age, (years)	0.049977	0.053542
Exertional angina pectoris		−0.746158
Prior myocardial infarction	0.441229	
Prior stroke or transient ischemic attacks	0.529301	
Diabetes mellitus		0.421626
Previous antiplatelet agent use	−0.502692	
Previous statin use		1.100651
**At admission**		
Arrhythmia	2.385515	2.014929
Systolic blood pressure, (mmHg)	−0.015563	
Diastolic blood pressure, (mmHg)	0.019416	−0.013703
Heart rate, (beats/min)	0.010054	
Killip class	0.521735	0.685218
Elevated biomarkers	1.214430	1.467987

* Age, blood pressure, heart rate and Killip class were modeled as continuous variables. Elevated biomarkers were binary variables.

Supplementary Table S8. Patient and procedural characteristics for all patients and subgroups based on gender.

Characteristics	Total	Male	Female	p
No. of patients	N=19237	N=14635 (70.1)	N=4602 (23.9)	
Age	64.49±12.04	62.99±12.19	69.27±10.17	<0.001
Height	163.85±7.41	166.69±5.67	154.81±4.49	<0.001
Weight	65.35±10.55	68.03±9.70	56.83±8.41	<0.001
**Medical history**				
Hypertension	10680 (55.5)	7685 (52.5)	2995 (65.1)	<0.001
Diabetes mellitus	5264 (27.4)	3741 (25.6)	1523 (33.1)	<0.001
Hyperlipoproteinemia	1971 (10.2)	1493 (10.2)	478 (10.4)	0.718
Smoke	10567 (54.9)	10363 (70.8)	204 (4.4)	<0.001
Prior myocardial infarction	4257 (22.1)	3314(22.6)	943 (20.5)	0.002
Prior stroke or transient ischemic attacks	619 (3.2)	434 (3.0)	185 (4.0)	<0.001
Family history of coronary heart disease	724 (3.8)	573 (3.9)	151 (3.3)	0.049
Previous antiplatelet agent use	9136 (47.5)	7073 (48.3)	2063 (44.8)	<0.001
Previous statin use	5386 (28.0)	4142 (28.3)	1244 (27.0)	0.094
Symptoms of angina pectoris	5912 (30.7)	4541 (31.0)	1371 (29.8)	0.113
Cardiac Arrest	249 (1.3)	196 (1.3)	53 (1.2)	0.326
Shock	798 (4.1)	594 (4.1)	204 (4.4)	0.267
**ACS category**				<0.001
STEMI	7283 (37.9)	5835 (39.9)	1448 (31.5)	
NSTEMI	4012 (20.9)	2986 (20.4)	1026 (22.3)	
UA	7942 (41.3)	5814 (39.7)	2128 (46.2)	
**At admission**				
Heart rate, (beats/min)	77.30±16.11	77.27±16.13	77.40±16.03	0.303
Systolic blood pressure, (mmHg)	128.31±23.03	127.16±22.64	131.97±23.87	<0.001
Diastolic blood pressure, (mmHg)	76.48±14.30	76.94±14.25	74.99±14.36	<0.001
Killip class				<0.001
Ⅰ	16968(88.2)			
Ⅱ	1224(6.4)			
Ⅲ	331(1.7)			
Ⅳ	714(3.7)			
ST elevation or depression	7028 (36.5)	5465 (37.3)	1563 (34.0)	<0.001
Arrhythmia	7514 (39.1)	5780 (39.5)	1734 (37.7)	0.028
TPN-T	1376.63±2595.71	1448.37±2655.80	1190.29±2384.30	<0.001
CERA	1.07±0.84	1.11±0.85	0.95±0.78	<0.001
Coronary artery blockage ≥50%	14248 (74.1)	11028 (75.4)	3220 (70.0)	<0.001
**In-hospital mortality**	414 (2.2)	288 (2.0)	126 (2.7)	0.002

ACS = acute coronary syndrome; (N)STEMI = (Non) ST-segment elevation myocardial infarction; UA = Unstable angina; TPN-T = cardiac troponin-T; CERA = creatinine.

Supplementary Table S9. Risk model discrimination and calibration performance for all patients and subgroups based on gender.

Characteristics	Total	Male	Female
No. of patients	N=19237	N=14635 (70.1)	N=4602 (23.9)
In-hospital mortality	414 (2.2)	288 (2.0)	126 (2.7)
**Risk model discrimination**			
GRACE	0.926 (0.911-0.940)	0.926(0.909-0.942)	0.925(0.897-0.9453)
GRACE 2.0	0.920 (0.905-0.935)	0.922(0.904-0.940)	0.914(0.884-0.944)
ACTION	0.945 (0.933-0.957)	0.944(0.930-0.957)	0.951(0.926-0.975)
TIMI	0.811 (0.787-0.835)	0.816(0.788-0.844)	0.796 (0.750-0.841)
CPACS	0.841 (0.821-0.861)	0.841(0.818-0.865)	0.837(0.799-0.874)
**Statistical comparison**			
GRACE vs. GRACE 2.0	P = 0.1480	P = 0.4137	P = 0.0772
GRACE vs. ACTION	P = 0.0004	P < 0.0042	P = 0.0250
GRACE vs. TIMI	P < 0.0001	P < 0.0001	P < 0.0001
GRACE vs. CPACS	P < 0.0001	P < 0.0001	P < 0.0001
GRACE2.0 vs. ACTION	P < 0.0001	P = 0.0008	P = 0.0048
GRACE2.0 vs. TIMI	P < 0.0001	P < 0.0001	P < 0.0001
GRACE2.0 vs. CPACS	P < 0.0001	P < 0.0001	P < 0.0001
ACTION vs. TIMI	P < 0.0001	P < 0.0001	P < 0.0001
ACTION vs. CPACS	P < 0.0001	P < 0.0001	P < 0.0001
TIMI vs. CPACS	P = 0.0077	P = 0.0425	P = 0.0932
**Risk model calibration – Mean risk prediction**			
GRACE	2.15±7.55	1.97±6.95	2.74±9.28
GRACE 2.0	2.15±7.99	1.97±7.41	2.74±9.64
ACTION	2.15±8.52	1.97±7.75	2.74±10.74
TIMI	2.15±4.51	1.97±4.37	2.74±4.88
CPACS	2.15±4.22	1.93±4.00	2.74±4.78
**Risk model calibration- Hosmer-Lemeshow**			
GRACE	P = 0.359	P = 0.725	P = 0.005
GRACE 2.0	P < 0.001	P < 0.001	P = 0.008
ACTION	P = 0.013	P = 0.230	P = 0.016
TIMI	P = 0.508	P = 0.600	P = 0.549
CPACS	P = 0.148	P = 0.100	P = 0.113

Supplementary Figure S1. Discrimination for the five risk models in subgroup analysis based on acute coronary syndrome category.

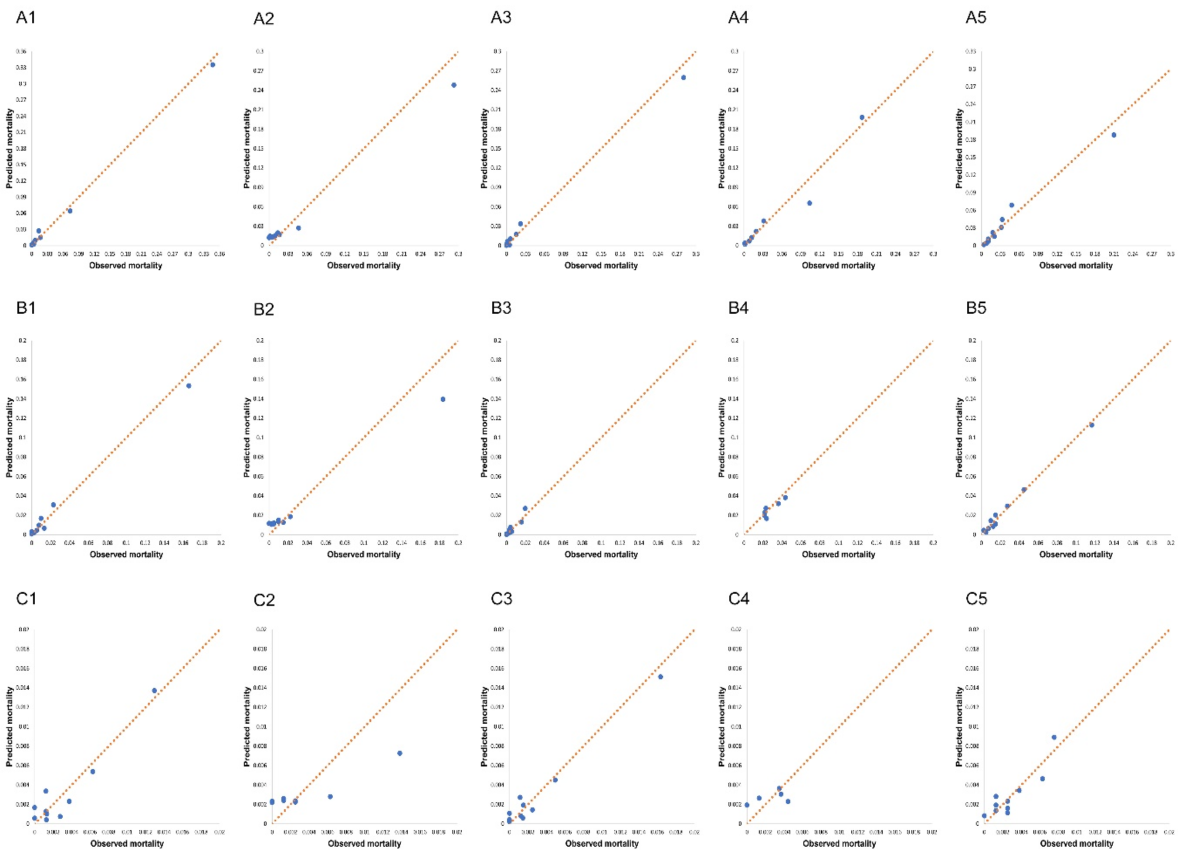

Risk model calibration for GRACE (1), GRACE 2.0(2), ACTION (3), TIMI (4) and CPACS (5) risk models, comparing observed and predicted mortality in risk quintiles of subgroup analysis based on acute coronary syndrome category, with STEMI (A), NSTEMI (B) and UA (C), respectively. (N)STEMI = (Non) ST-segment elevation myocardial infarction. UA = Unstable angina.

Supplementary Figure S2. Discrimination for the five risk models in subgroup analysis based on gender.

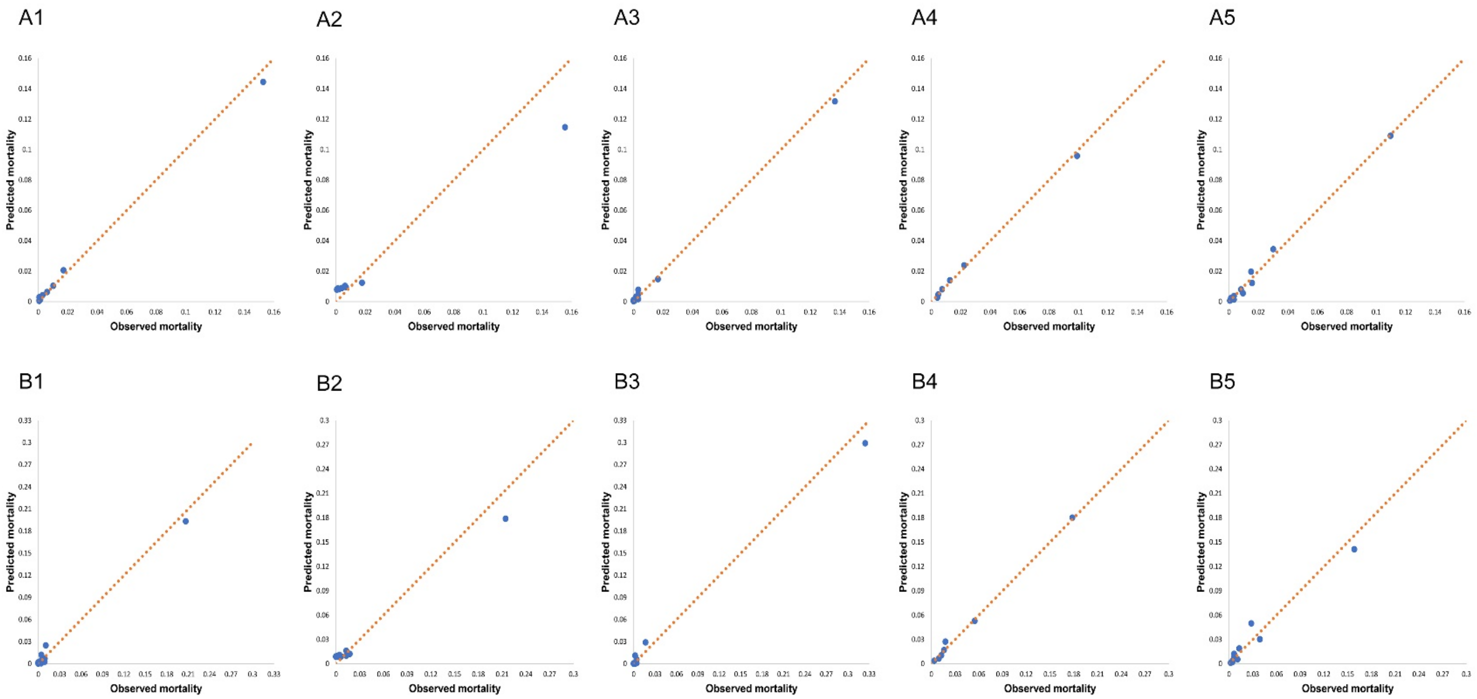

Risk model calibration for GRACE (1), GRACE 2.0(2), ACTION (3), TIMI (4) and CPACS (5) risk models, comparing observed and predicted mortality in risk quintiles of subgroup analysis based on gender, with Male (A) and Female (B), respectively.
